# Fetal and Neonatal Endocrine Disruptors

**DOI:** 10.4274/Jcrpe.569

**Published:** 2012-06-09

**Authors:** Tolga Ünüvar, Atilla Büyükgebiz

**Affiliations:** 1 T.C. Ministry of Health, İstanbul Kanuni Sultan Süleyman, Training and Research Hospital, Department of Pediatric Endocrinology, İstanbul, Turkey; 2 Bilim University Faculty of Medicine, Department of Pediatric Endocrinology, İstanbul, Turkey; +90 212 543 62 70 +90 212 571 47 90tunuvar@lycos.com

**Keywords:** endocrine disruptors, fetal, neonatal

## Abstract

Endocrine disruptors are substances commonly encountered in every setting and condition in the modern world. It is virtually impossible to avoid the contact with these chemical compounds in our daily life. Molecules defined as endocrine disruptors constitute an extremely heterogeneous group and include synthetic chemicals used as industrial solvents/lubricants and their by-products. Natural chemicals found in human and animal food (phytoestrogens) also act as endocrine disruptors. Different from adults, children are not exposed only to chemical toxins in the environment but may also be exposed during their intrauterine life. Hundreds of toxic substances, which include neuro-immune and endocrine toxic chemical components that may influence the critical steps of hormonal, neurological and immunological development, may affect the fetus via the placental cord and these substances may be excreted in the meconium. Children and especially newborns are more sensitive to environmental toxins compared to adults. Metabolic pathways are immature, especially in the first months of life. The ability of the newborn to metabolize, detoxify and eliminate many toxins is different from that of the adults. Although exposures occur during fetal or neonatal period, their effects may sometimes be observed in later years. Further studies are needed to clarify the effects of these substances on the endocrine system and to provide evidence for preventive measures.

**Conflict of interest:**None declared.

## INTRODUCTION

Endocrine disruptors (xenohormones) are exogenous substances or compounds that cause adverse health effects in the organism by disrupting the endocrine functions (1,2,3).Endocrine disruptions mostly occur on the basis of genetic predisposition. Natural or synthetic chemical endocrine disrupting substances that we are surrounded with and are exposed to in the modern world lead to clinical disorders by intervening with hormonal physiology (4). At first, endocrine disruptors were thought to act only via nuclear hormone receptors such as estrogen, androgen, progesterone, thyroid and retinoid receptors. However, recent studies have shown that the mechanism of action of endocrine disruptors is not that simple. Endocrine disruptors are thought to exert effects on endocrine and reproductive systems through nuclear receptors, non-nuclear steroid hormone receptors (membrane estrogen receptors), non-steroid receptors (neurotransmitter receptors such as serotonin, dopamine, norepinephrine receptors), orphan receptors (aryl hydrocarbon receptor), enzymatic pathways involving steroid biosynthesis and/or metabolism, and numerous other mechanisms (3). Endocrine disruptors are also known as endocrine modulators, hormone active agents, endocrine active agents, endocrine toxins or xenohormones (5,6). Molecules defined as endocrine disruptors constitute an extremely heterogeneous group and include synthetic chemicals used as industrial solvents/lubricants and their by-products (polychlorinated biphenyls, polybrominated biphenyls, dioxins), plastics (bisphenol A), plasticisers (phthalates), pesticides [methoxychlor, chlorphyrifos, dichlorodiphenyl-trichloroethane (DDT)], fungicides (vinclozolin) and pharmaceutical agents (diethylstilbestrol) (3). Natural chemicals found in human and animal food (phytoestrogens such as genistein, coumestrol) also act as endocrine disruptors (5,7) (Table 1). Different from adults, infants and children are not exposed only to chemical toxins in the environment but may also be exposed indirectly during their intrauterine life. Hundreds of toxic substances may affect the fetus via the placental cord and these substances may be excreted via the meconium. These include countless number of neuro-immune and endocrine toxic chemical components that may influence the critical steps of hormonal, neurological and immunological development. It has been demonstrated in animal and human studies that affected offsprings are not only born with congenital abnormalities but may also suffer from several health and behavioral problems throughout their lifespan (8). Measurement of endocrine disruptor levels in the umbilical cord blood of newborns is one of the several methods to determine intrauterine fetal exposure to chemical substances. In a study conducted in the US by the Environmental Working Group (EWG), 413 toxic substances were sought in the umbilical cord blood of 10 newborns, with positive results for 287 of these substances (Table 2). In the same study, many endocrine-disrupting chemical compounds including pharmaceutical agents, illegal drugs, heavy metals and pesticides were also detected in the meconium samples from the newborns. Children and especially newborns are more sensitive to environmental toxins compared to adults. Metabolic pathways in infants are immature, especially in the first months of life. Newborns differ from adults in their ability to metabolize, detoxify and eliminate many toxins. It has been demonstrated in various studies that newborns are affected from chemical toxins to a greater degree than adults (9). Although exposures have occurred during fetal life or during the neonatal period, the effect of these exposures may sometimes not be observed for many years. For these reasons, some of the states caused by endocrine disruptors are among examples of adult disease states of fetal origin (10). Owing to the fact that endocrine disruptors have low water solubility and high lipid solubility, they accumulate in fatty tissues. Thus, their long-term effects may be observed in later years.

**Effects of Endocrine Disruptors on Growth**

Intrauterine exposure to endocrine disruptors has been associated with low birth weight, low height and low head circumference in the newborns. The main chemical compounds that affect growth in infancy are the chlorinated pesticide metabolites dichlorodiphenyldichloroethylene (DDE), organophosphate pesticides and polycyclic aromatic hydrocarbons (PAH). Growth retardation has been noted in a 5-year follow-up of children after prenatal exposure to dioxins polychlorinated dioxins and furans (PCDD/PCDFs) (11).

**Polycyclic Aromatic Hydrocarbons **

These substances are found in combustion by-products associated with smoking, vehicle exhaust, factory chimneys, forest fires, and jet motors. It is well known that maternal smoking during pregnancy is associated with low birth weight (12). It has also been shown that air pollution in the cities is another source of PAH and that it is associated with low birth weight, reduced height and low head circumference in the newborn (13,14).

**Organophosphate Pesticides**

It has been reported that intrauterine exposure to these substances is associated with height, weight and head circumference retardation in the newborns (15). Moreover, severe mental retardation, as well as abnormalities pertaining to the development of the brain, eyes, ears, teeth, heart, feet, breast and sexual organs (undescended testicles, microphallus, labial fusion) have been observed in affected infants at birth (16).

**Effects of Endocrine Disruptors on the Development of the Immune System**

Some lead-containing endocrine-disrupting compounds have been shown to result in domination of T helper cells (TH2) in the fetal immune system, which in turn leads to an increased predisposition to asthma and allergies in newborns and infants (17). It has been shown that intrauterine exposure to dioxin leads to the development of autoimmunity in animals (18). Since most of the developmental activity in the immune system occurs in the first trimester of pregnancy, exposure to foreign substances during this period may have a greater impact. Depending on the time of impact, exposure to PAH may lead to imbalances in the immune system such as decrease in T cells, increase in B cells, and elevation in IgE levels (19). It has been found in animal studies that intrauterine exposure to polychlorinated biphenyls (PCBs) results in atrophy of the thymus. Respiratory tract infections have been noted to be more common and to show a tendency to recur more frequently in children exposed to PCB and DDE during the prenatal period as compared to children who are not affected (20,21). In addition to increased tendency for infections in infants affected by endocrine disruptors, immune response is also weakened. It was demonstrated in a Dutch study that insufficient amounts of antibodies were formed following measles-mumps-rubella vaccine in children exposed to PCB (21).

**Effects of Endocrine Disruptors on the Nervous System**

As the blood-brain barrier has not yet completed its development during the fetal development of the nervous system, the fetus is more sensitive to all neurotoxins.

**Mercury:** High levels of mercury have been found in hair samples of infants born to women who have consumed high amounts of seafood during their pregnancy. These children may experience cognitive and behavioral disorders in later life (22).

**Lead:** Reduction in memory and problem-solving abilities have been noted in rats with prenatal lead exposure (23). Deficits in mental abilities have been reported in a 24-month follow-up of children with intrauterine exposure to high amounts of lead from maternal bone stores (24).

**Polychlorinated biphenyls:** Long-term neurological deficits and persistent behavioral problems have been reported in rats with neonatal PCB exposure. Also, intellectual functions have been found to be affected in infants whose mothers had consumed fish exposed to PCB during pregnancy (25). Reduction in comprehension and cognitive functions have been noted even in children born as late as six years after the incident in which their mothers were affected and these children were also observed to have significantly more behavioral and activity problems compared to the control group (26). 

**Organophosphate pesticides:** Children with intrauterine exposure to organophosphate pesticides have been found to exhibit abnormal reflexes at the time of birth (27). These children were also noted to have poor short-term memory, slow response times as well as impaired mental development (28).

**Effects of Endocrine Disruptors on the Urogenital System**

Sex steroids, and especially androgens are very important for normal intrauterine sexual development (29). Sexual differentiation of a male fetus is androgen-dependent (estrogen is also effective in a small degree). However, sexual differentiation of a female fetus is often independent of androgens and estrogens. Therefore, sexual development disorders related to endocrine disruptors, which excellently mimic estrogens and/or block androgens, lead to different clinical presentations in girls and boys (30) (Table 3).

**The Male Urogenital System**

A weak association has been found between maternal serum concentrations of PCB, dichlorodiphenyltrichloroethane (DDT) and DDE (primary DDT metabolites) and occurrence of undescended testicles and hypospadias in infants born to these mothers (31,32). On the other hand, significant relationships between development of hypospadias or undescended testicle and consanguineous marriages or pesticide exposure have been reported (33). It has also been shown that 2,3,7,8-tetrachlorodibenzo-p-dioxin (TCDD) exposure of male rats during intrauterine and lactation periods leads to sexual differentiation in the brain and sexual behavior changes (34,35). Estrogen and testosterone levels in umbilical cord blood of newborns whose mothers were exposed to high levels of PCDD and PCDF were found to be lower compared to the control group (36). In a study conducted on Taiwanese boys exposed to intrauterine PCB and PCDF, a reduction in serum testosterone levels together with an increase in serum follicle-stimulating hormone (FSH) and estradiol levels during puberty were reported (37). It has been shown in experimental studies that phthalates have antiandrogenic and mild estrogenic effects and may cause ambiguous genitalia in male infants (38). The effects of certain specific endocrine disruptors on male urogenital system are presented in Table 4.As the half-life of organochlorines is very long (years or even decades), intrauterine exposure should be suspected in children of prepubertal or pubertal ages admitted to hospital with genital abnormalities. Further studies are needed to clarify the effects of endocrine disruptors on the development of the male urogenital system (39).

**The Female Urogenital System**

**Dioxins:**Although not classified as xenoestrogens, dioxins may lead to estrogenic or antiestrogenic effects depending on time of exposure, site (organ) of exposure and presence of estrogen. It has been demonstrated that tumor incidence is increased and latent period of tumor development is decreased in rats exposed to TCDD on the 15^th^ day of gestation and to the chemical carcinogen dimethylbenzanthracene (DMBA) on the 50th day of gestation (40). There have also been studies reporting that fetal TCDD exposure increases breast cancer risk and leads to deterioration in breast tissue morphology (41). Kakeyama et al (42) reported that exposure to low-dose TCDD during the prenatal period led to early activation of the hypothalamic-pituitary-gonadal axis and precocious puberty in rats. 

**Bisphenol A:** Bisphenol A (BPA), which is a xenoestrogen, is a chemical endocrine disruptor frequently encountered in every setting of daily life. While maternal exposure to BPA may indirectly affect the fetus, it may also affect the newborn directly through infant formulas, breast milk and preserved foods (43). In fact, high levels of BPA have been measured in human placental tissue as well as in maternal and fetal plasma at the time of birth (44). Urinary BPA levels measured in children and adolescents have been reported to be higher compared to adults (45). BPA has also been detected in the breast milk of lactating mothers. In the light of these findings, it can be suggested that human fetuses and newborns may readily be exposed to this chemical. Recent studies have reported that food is not the only source of BPA exposure and that parenteral applications may also be a source. BPA half-life in humans is much longer than expected (46). Morphological and developmental abnormalities were noted in the breast tissue of fetuses of mother rats exposed to BPA for 14 days starting from the 8^th^ day of gestation. Mammary duct epithelial cell proliferation in these fetuses was found to be significantly greater compared to that in fetuses whose mothers were not exposed to BPA (47). It has also been observed that intraductal hyperplasias that are defined as preneoplastic lesions may appear in the 3rd postnatal month subsequent to this proliferation and increased ductal branching (48). In another study, 3-4-fold increase in precancerous lesions (intraductal proliferation) during puberty and adulthood has been shown to occur in rats as a result of fetal BPA exposure (49). These results indicate that significant prenatal exposure to BPA leads to persistent changes in breast tissue structure and development, precancerous lesions and carcinoma in situ. Exposure to BPA and other chemicals with estrogen-like effects has been listed as one of the reasons for the increased incidence of breast cancer in modern societies in recent years. 

**Polychlorinated compounds:** Yang et al (50) reported that prenatal exposure to PCBs and PCDFs results in abnormal menstruations and very high FSH and estradiol levels in the follicular phase of the cycle in adolescent girls aged between 13 and 19 years. In a Chinese study, prenatal phthalate exposure (di-n-butyl phthalate (DBP) and di-2-ethylhexyl phthalate (DEHP) was shown to increase the risk of low birth weight (51). 

**Diethylstilbestrol:**Epidemiological studies suggest that exposure to xenoestrogens, such as diethylstilbestrol (DES) and DDT, during the fetal period and during puberty increases the risk of developing cancer. In several studies, it has been reported that intrauterine DES exposure may cause cervical uterine and fallopian tube abnormalities, subfertility, infertility and ectopic pregnancies (52). The effects of certain specific endocrine disruptors on the female urogenital system are presented in Table 5. In newborns with intrauterine growth retardation and in those with sexual differentiation disorders such as hypospadias and undescended testicle, breast milk and maternal serum should be screened for endocrine disruptors and these biological samples should be preserved for further research. Research priority should be given to the identification of early markers and indicators of endocrine disruptors encountered in fetal life (39). 

**Effects of Endocrine Disruptors on Thyroid Function**

**PCBs:**Although PCBs have been prohibited in many countries for years due to their very strongly lipophilic features, they can still be detected in human and animal tissue samples (5,53). PCB, especially their biologically active hydroxylated metabolites, has great structural similarity with thyroxine (T4). In animal studies, it has been shown that intrauterine PCB exposure has negative impact on thyroid hormone levels by leading to a decrease in total T4, free T4 (fT4) and total triiodothyronine (T3) and an increase in thyrotropin (TSH) level and that these effects are dose-dependent (54). There is strong evidence demonstrating reduced thyroid hormone levels in infants whose mothers experienced perinatal exposure to PCB or its hydroxylated metabolites (5,55,56,57). Antibodies against thyroid peroxidase and an increase in thyroid volume have also been noted in the follow-up of infants exposed to PCB (58,59). In another study, PCB levels measured in breast milk have been shown to correlate with low postpartum maternal thyroid hormone levels and high fetal TSH levels at postnatal 2nd-3rd months (54,60).

**Dioxins:** PCDDs and PCDFs are permanent and very toxic environmental pollutants that are formed as by-products from destruction of several chemical substances, including substances used in pesticide production and for bleaching of cellulose in paper production. These compounds initially diffuse into air, then into soil and then contaminate meat, fish, dairy products and breast milk. When pregnant rats were exposed to PCDD, in male offspring rats, the T4 levels were found to be decreased, while an increase was noted in TSH levels (61). Five-year follow-up revealed a positive correlation between serum thyroid hormone levels and PCDD/PCDF levels in children with prenatal exposure to dioxins (11).

**Polybrominated diphenyl ethers:** These substances are found in several materials used in daily life including plastic coating of electronic devices such as television and computer, in light bulbs, car spare parts, carpets and bedspreads, dye substances and synthetic textile products. Tetrabromobisphenol A (TBBPA) and polybrominated biphenyls are also classified within the same group as polybrominated diphenyl ethers (PBDE). TBBPA and PBDE show a closer structural similarity with thyroid hormone than PCB (53). Prenatal and postnatal thyroid hormone levels have been found to be low in rats with perinatal exposure to PBDE (62). Even low doses of PBDE have affected thyroid functions; T4 levels of offspring of pregnant rats exposed to PBDE have been found to be decreased at postnatal 3^rd^ week (63).

**Pesticides:** DDT, hexachlorobenzene (HCB) and nonylphenol are the chemicals whose effects on thyroid functions have been investigated most frequently. Although these substances have been prohibited in many countries, they can be still detected in the environmental cycle as they are still being used in some countries and they have very long half-lives. Evidence regarding the disrupting effects of pesticides on thyroid hormones have been noted in numerous animal and toxicological studies (53).

**Perfluorinated chemicals:** Owing to their surface protecting features, perfluorinated chemicals (PFCs) are chemicals that are very frequently used in daily life. T4 levels have been found to be decreased in both pregnant rats and their offspring as a result of short- and long-term exposure to these substances (64,65). A transient increase in T4 and a decrease in TSH followed by a decrease in T4 and T3 have been noted following exposure to a single dose of PFC (66).

**Phthalates:** Phthalates are often impossible to avoid since they are widely present as additives in many plastic products and in several industrial and commercial products. They are found in soft plastic toys, floor coverings, home cleaning products, medical devices, blood bags, cosmetic products and air cleaners. As phthalates are water-soluble, they can reach the fetus via the amniotic fluid in pregnant mothers. Particularly newborns in contact with materials such as catheters and medical tubings in hospitals are exposed to phthalates and they may subsequently develop transient thyroid dysfunction. T3 and T4 levels have been found to be decreased in rats exposed to phthalates in a dose-dependent manner (67). Histopathological changes in the thyroid gland have been noted in some studies (68). Also, a negative correlation has been found between phthalate exposure and total T4 and fT4 levels in pregnant women (69).

**BPA:** BPA is a chemical that we are easily exposed to because it is found within the structure of many plastic products including food containers, plastic bottles, feeding bottles, and inner parts of cans. T4 levels have been found to be increased at postnatal 15 days in offspring of pregnant rats exposed to BPA (70). However, there have also been studies showing that BPA does not affect thyroid functions (71). Further studies are needed to evaluate the harmful effects of several groups of chemical disruptors on thyroid functions.

**Effects of Other Compounds as Endocrine Disruptors**

In addition to those mentioned above, effects of other chemical compounds on the endocrine system have also been observed. Children with intrauterine exposure to HCB, a type of chlorine pesticide, were found to have a tendency for obesity when they reach 6 years of age, and they have been shown to have a 2.5-3-fold increased risk of being overweight compared to children not exposed to HCB, independent from diet type and daily activity (72). Testicular dysgenesis syndrome, a clinical entity which comprises testicular cancer, urogenital abnormalities and reduced semen quality, is thought to occur by contact of the fetal testis with endocrine disruptors during intrauterine development (73). Phytoestrogens are abundantly present in our daily food, and a 500 times greater estrogenic effect has been found in infants fed by soy-based formulas as compared to those fed by cow’s milk-based formulas (74). Genistein, found in soy beans, is a weak estrogenic substance which may lead to several pathologies in the reproductive system and also in the thyroid gland through thyroid peroxidase inhibition (75). The effects of intrauterine exposure to endocrine disruptors, which can be detected in umbilical cord blood and meconium samples, are summarized in Table 6.

## CONCLUSION

Endocrine disruptors are commonly found substances that we may encounter in every setting and condition in the modern world and it is virtually impossible to avoid the contact with these chemical compounds in our daily life. Epidemiological and toxicological studies that have been performed over the years and have provided scientific data for the protection of human health and wildlife have shown that nature as a whole is under risk due to these chemicals. These studies also provide evidence to inform local authorities about this threat and about preventive measures to be taken on the issue of endocrine disruptors, particularly with regard to pregnant women and children. These local measures should be considered as an initial step in prevention and global awareness studies should be performed in addition to local studies. Further animal and human studies are also needed to investigate the effects of intrauterine endocrine disruptor exposure on adult health.

## Figures and Tables

**Table 1:**
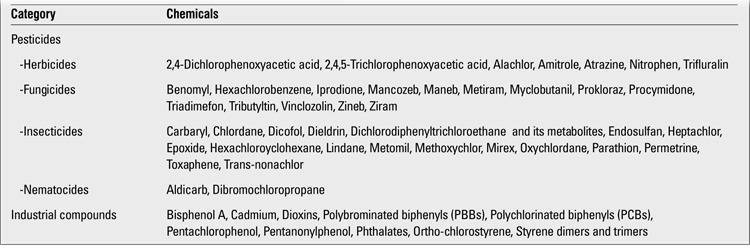
The most common chemicals found in the environment ([ref:5]5[/ref],[ref:7]7[/ref])

**Table 2:**
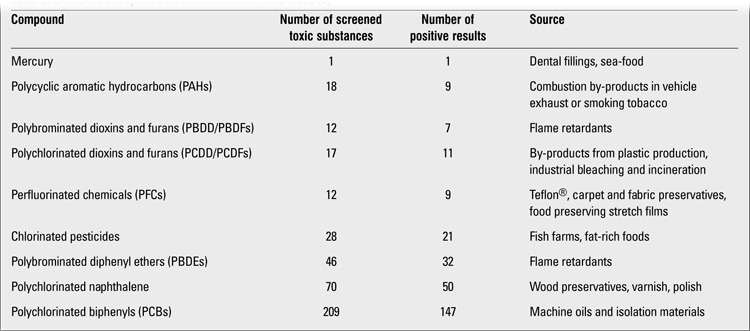
Endocrine disruptors detected in umbilical cord blood ([ref:8]8[/ref])

**Table 3:**
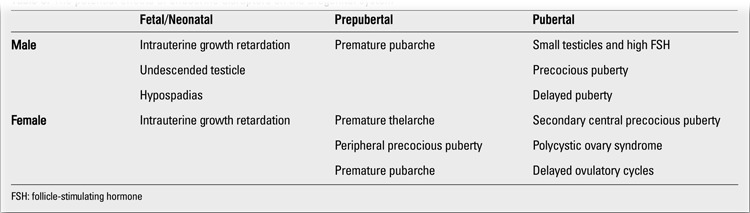
The potential effects of endocrine disruptors on the urogenital system

**Table 4:**
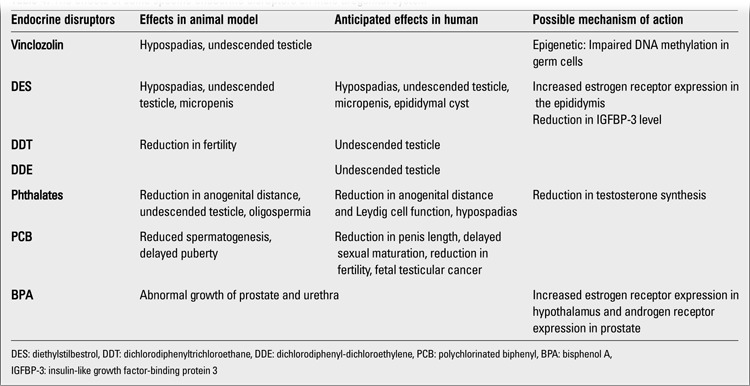
The effects of some specific endocrine disruptors on male urogenital system

**Table 5:**
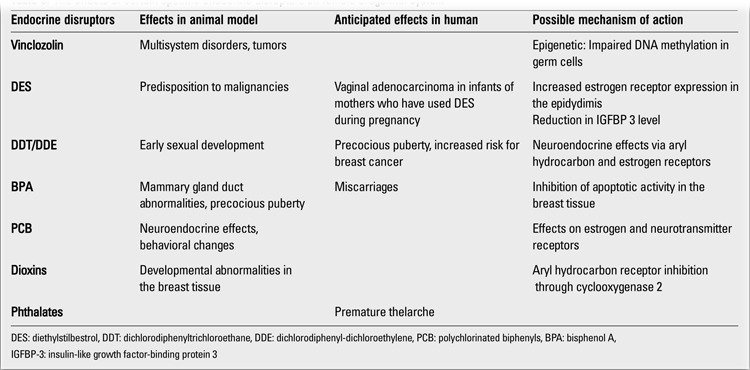
The effects of certain specific endocrine disruptors on female urogenital system

**Table 6:**
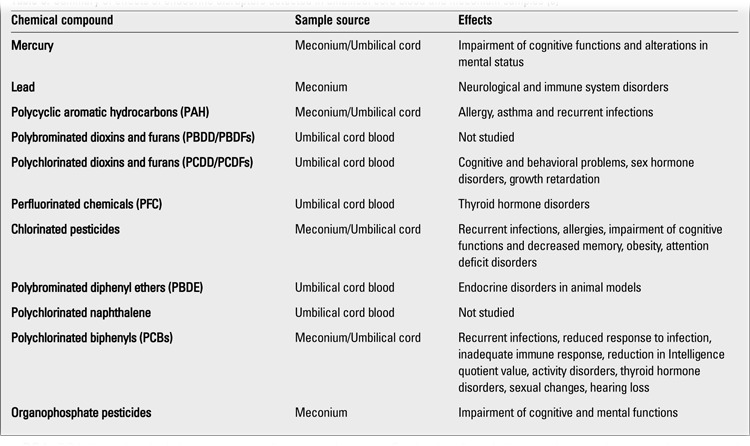
Summary of effects of endocrine disruptors detected in umbilical cord blood and meconium samples (8)
